# Water splitting by MnFe_2_O_4_/Na_2_CO_3_ reversible redox reactions†

**DOI:** 10.1039/d2ra05319e

**Published:** 2022-11-02

**Authors:** Yimin Deng, Shuo Li, Raf Dewil, Lise Appels, Miao Yang, Huili Zhang, Jan Baeyens

**Affiliations:** KU Leuven, Department of Chemical Engineering, Process and Environmental Technology Lab 2860 Sint-Katelijne-Waver Belgium Jan.Baeyens@kuleuven.be; Beijing University of Chemical Technology, Beijing Advanced Innovation Centre of Soft Matter Science and Engineering 100029 Beijing China; University of Oxford, Department of Engineering Science Parks Road Oxford OX3 3PJ UK; Beijing University of Chemical Technology, School of Life Science and Technology 100029 Beijing China

## Abstract

Future energy systems must call upon clean and renewable sources capable of reducing associated CO_2_ emissions. The present research opens new perspectives for renewable energy-based hydrogen production by water splitting using metal oxide oxidation/reduction reactants. An earlier multicriteria assessment defined top priorities, with MnFe_2_O_4_/Na_2_CO_3_/H_2_O and Mn_3_O_4_/MnO/NaMnO_2_/H_2_O multistep redox cycles having the highest potential. The latter redox system was previously assessed and proven difficult to be conducted. The former redox system was hence experimentally investigated in the present research at the 0.5 to 250 g scale in isothermal thermogravimetry, an electrically heated furnace, and a concentrated solar reactor. Over 30 successive oxidation/reduction cycles were assessed, and the H_2_ production efficiencies exceeded 98 % for the coprecipitated reactant after these multiple cycles. Tentative economics using a coprecipitated reactant revealed that 120 cycles are needed to achieve a 1 € per kg H_2_ cost. Improving the cheaper ball-milled reactant could reduce costs by approximately 30 %. The initial results confirm that future research is important.

## Abstract 2

Investigating H_2_ production by MnFe_2_O_4_/Na_2_CO_3_/H_2_O redox cycles, using different reactants. Using the more efficient coprecipitated reactant, production costs will be ∼1€ per kg H_2_, if 120 cycles are achieved. Improving the cheaper ball-milled reactant is recommended.

## Graphical abstract

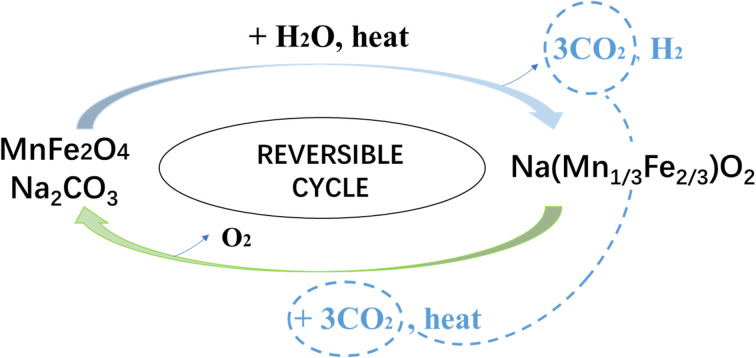

## 1. Introduction

The future annual worldwide need for H_2_ was estimated by Liu *et al.*:^1^ reducing global CO_2_ emissions requires that at least 10% of hydrocarbons (currently 14 billion tons of oil equivalent^2^) be substituted by alternative renewable energy resources, including “green” H_2_. If only H_2_ is considered as a substitute energy carrier, approximately 1000 Mtons of nonfossil-based H_2_ are needed. Current H_2_ production is more than 10 times below the required 10% replacement target,^3^ but electrolysis using renewable energy is promoted. Neither hydropower^3^ nor nuclear energy are sufficient to meet this target.^4^ The nuclear energy production was only 25 exajoule (even less than 39 exajoule per year of hydropower). The negative public attitude towards nuclear energy and the limited reserves of uranium will probably not foster increased nuclear power.

Currently, green electrolytic H_2_ can be produced using mostly wind or photovoltaic electricity. Its production requires an average electricity consumption of approximately 50 kW h kg^−1^ H_2_ at an overestimated efficiency of 80%.^5^ The 2019 global renewable energy production, without hydro-energy, was estimated at 2800 TW h. If these sources were devoted to solely produce H_2_, only 56 Mtons of electrolysis H_2_ could be generated, and this at a 2- to 4-fold production cost of the traditional petrochemical methods. Renewable technologies are therefore unable to meet the 1000 Mtons H_2_ goal.

It is therefore expected that fossil hydrocarbons will remain the important H_2_ sources in the near future, despite their environmental impact through CO_2_ emissions. Steam methane reforming yields approximately 10 kg of CO_2_ per kg H_2_, and is hence classified as “grey hydrogen” production method.^3^ The application of carbon capture and storage could make this technology “cleaner”, but involving significant costs to nearly double the price of the produced H_2_.^6^

Within the “green” H_2_ production methods,^3^ thermochemical water splitting by redox systems^5^ has a considerable potential, and is the subject of the present research. Alternative methods were, however, also investigated.^7,8^

### 1.1 H_2_ from multicycle thermal redox water splitting

Thermochemical redox water splitting reactions are candidates for H_2_ production. Water is abundant, inexpensive, and its decomposition or subsequent combustion is free from CO_2_ emissions. Thermochemically splitting water into H_2_ and O_2_ involves different reaction pathways, but the overall endothermic reaction (Δ*H* = 286 kJ mol^−1^) is H_2_O → H_2_ + ½O_2_.

These thermochemical reactions to produce H_2_ offer major advantages in comparison with common alkaline water electrolysis, which has a low efficiency of approximately 20% (30% for electricity and 65% for electrolysis). The high-temperature thermochemical H_2_ production efficiency is much higher, provided cheap renewable or waste high-temperature heat is available.

These water splitting systems have been proposed since 1964. The initial vanadium–chlorine cycle was, however, abandoned for its low efficiency, high cost, and the formation of toxic and hazardous products. The cycles were however further developed, and approximately 25 thermochemical cycles are currently proposed. Deng *et al.* examined and ranked these proposed redox reactions by multiple criteria assessment, including quantified parameters of thermal, chemical, environmental and economic nature.^5^

Very high temperature reactions (≫ 1000 °C) of metal–metal oxides/hydroxides, doped ceria, or perovskites were not considered since such high temperatures would necessitate the application of high-cost alloys to construct redox reactors. Selected redox systems should operate at temperatures that would limit reactor wall temperatures below 1000 °C, thus operating the redox material bed at maximum 800 °C.^5^ Based on this important target, 4 out of 24 redox reactions were finally selected, and included MnFe_2_O_4_/Na_2_CO_3_, Mn_3_O_4_/MnO/NaMnO_2_, U_3_O_8_/UO_2_CO_3_ and ZnO/Fe_3_O_4_/ZnFe_2_O_4_. The U_3_O_8_ cycles were discarded due to nuclear hazards. The ZnO cycles scored significantly lower than both remaining redox systems, and were not further investigated. The Mn_3_O_4_/MnO/NaMnO_2_ four-step redox process was assessed, but required an operation temperature of approximately 800 °C and suffered from poor reversibility of the redox cycles.^1^ The MnFe_2_O_4_/Na_2_CO_3_ cycle remained the selected system under scrutiny.

### 1.2 Solar heat-induced redox systems

The use of solar energy in the redox cycles for H_2_ production was previously advocated.^9,10^ Metal oxide redox pairs are considered the simplest, and the most environmentally friendly. Solar heat supplies the sensible and reaction heats of the oxidation and reduction cycles. The ferrite system was shown to split water at low temperatures in the oxidation reaction and reduce CO_2_ in the reduction reaction.^11–13^ Solar-induced two-step water splitting by (Ni, Mn) ferrite and ZnO/MnFe_2_O_4_ was achieved at approximately 1000 K.^14^ The solar-based ZrO_2_-supported Co(ii)-ferrite cycle was finally tested but the required temperatures of 1000 °C for the oxidation and 1400 °C for the reduction, and the attrition of the reactant during the reduction were prohibitive for its further application. Carbon-bearing systems failed in repeated cycles.^12,13^

### 1.3 Literature findings on MnFe_2_O_4_ and its applications

Spinel ferrites (MnFe_2_O_4_) are a significant class of magnetic materials, with excellent electrical properties. Their development is therefore mainly linked with electronic uses, for example shifters, frequency transformers, PCs, TV, cell phones, adsorbents, among other applications. Their use in water splitting was launched during the past decade.

The manufacturing methods are diverse, with main objectives to produce MnFe_2_O_4_ particles of small size, high crystallinity, and large specific surface area. The comparison of the manufacturing methods and the relevant physicochemical properties is given in Table 1. The manufacturing methods used in the present research, *i.e.* by simple ball-milling and by co-precipitation, are included in the table.

**Table 1:** Comparison of physicochemical properties of MnFe_2_O_4_ particles synthesized *via* different methods

Synthesis method	Reaction *T* (°C)	Time (h)	Particle size (nm)	Surface area (m^2^ g^−1^)	Ref.
Polyol	210	3	7	165.39	15
Co-precipitation	70	2	20–80	84.5	16
	95	2	30	—	17
	50	3	24	—	18
	70	1	36	—	19
	75–80	6	14	0.293 (ref. 20)	21
	80	6	80	—	22
Sol–gel	70	2	45	—	19
Ball milled	900	1	6.78–8.06		23
Thermal decomposition	295	1	7	—	24
	270	1.5	18.9	—	25
Solution combustion	300	—	30–35	33	26
Hydrothermal	200	12	30–50	—	27
	220	10	14.5	—	28
	180	28	280	32.19	29
	180	16	16	—	19
Solvothermal	200	24	8.6	—	30
	180	12	60	70	31
	500–600	24	12–22	—	32
	200	8	250–260	—	33
Co-precipitation	80	2	40	132	This work
Ball milled	20	1	100	4.92	This work

Whereas ball-milled reactant appears less performance due to its coarse particle size and low surface area, coprecipitated reactant seems to meet the main property targets. Our coprecipitation differs from other cited synthesis methods through its use of metal chlorides as precursors, rather than the previously favoured metal nitrates or sulphates.

**Fig. 1:**
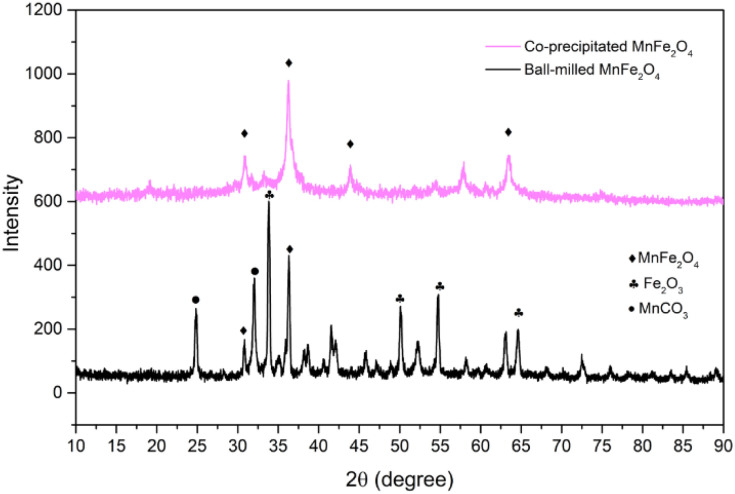
Diffractograms of the selected reactants.

The crystalline of both ball-milled and coprecipitated MnFe_2_O_4_, is determined from XRD analysis. The diffractograms of both reactants are shown in Fig. 1.

In both cases, diffraction peaks were matched with the respective references of the ICDD cards. The main peaks correspond to the space group *Fd*3̄*m* (spinel ferrite). The crystallite size for each phase was determined from the Sherrer's equation,^34^ accounting for the most intense XRD-peak. The crystal size, *δ*, is determined as:
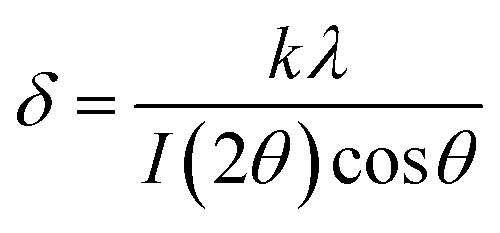
with, *k*, as a correction factor, commonly 0.9; *λ* as the wave length of X-ray diffraction of the α-Cu electrode (0.154 nm); and *θ* is the Bragg angle (degree). Therefore, the average crystallite size of ball-milled MnFe_2_O_4_ is determined to be about 15.5 nm, and the co-precipitated MnFe_2_O_4_ is about 10.4 nm. It can be noted that the crystallite sizes are very similar, despite the different synthesis method.

The fairly novel application in water splitting relies on the thermochemical cycle of MnFe_2_O_4_/Na_2_CO_3_, as described by the following reactions:^1^2MnFe_2_O_4_ (s) + 3Na_2_CO_3_ (s) + H_2_O (g) → 6Na(Mn_1/3_Fe_2/3_)O_2_ (s) + 3CO_2_ (g) + H_2_ (g)6Na(Mn_1/3_Fe_2/3_)O_2_ (s) + 3CO_2_ (g) → 2MnFe_2_O_4_ (s) + 3Na_2_CO_3_ (s) + 0.5O_2_ (g)

The optimal operation temperature of both reactions is approximately 700 to 750 °C.^35^ H_2_ production at lower temperatures shows slower kinetics, and complete regeneration cannot be fully accomplished.^36,37^ The reduction step is problematic and less effective, although adding Fe_2_O_3_ could help to overcome the problem.^38^ The reduction step seems to pose a main problem for all the redox pairs that were reported,^35,39^ and the reaction kinetics are not fully defined. The problems in the oxygen-releasing step are amplified along with the increasing experimental scale.

Although literature is still scarce, earlier research was reported by Murmura *et al.*,^39^ and by Varsano *et al.*^35^ Both Murmura *et al.*^39^ and Varsano *et al.*^35^ used about 40 mg MnFe_2_O_4_/Na_2_CO_3_ at the lab-scale for temperatures between 700 and 800 °C, or 600 and 800 °C, respectively. The H_2_ yield obtained after 1 h of oxidation varied from 81% (700 °C) to 86% (800 °C).

Varsano *et al.*^40^ repeated the tests in a 1 kW concentrated solar facility at 750 and 800 °C and obtained H_2_ yields of approximately 20 to 37% at 750 °C, and ∼72% at 800 °C. The lower H_2_ efficiency in the solar-driven reactor was attributed to a nonuniform temperature within the solar reactor, and to a too coarse particle size (0.5–2 mm) used in the solar reactor.^41^ Such coarse particles have a very low active surface area. In general, the previous studies paid insufficient attention to the individual steps (oxidation and reduction), to the reaction kinetics, and to the long-duration and multicycle operation.

### 1.4 Objectives of the research

The art-to-date studies of redox systems were mostly limited to single steps of the cycles, with experiments performed on the milligram scale, and without considering long-duration cyclability and process economics. The present research will for the first time assess water splitting on a small and a larger scale, by thermogravimetric analysis (TGA), in an isothermal electric furnace, and in a concentrated solar rig. Realistic operating conditions and equipment scale will be accounted for. The study is limited to the priority-selected MnFe_2_O_4_/Na_2_CO_3_ cycles.^1^ It is expected that the results will provide better insight into thermochemical H_2_ production in equipment of different scales. Special attention will be given to the results obtained in multiple cycles, hence providing a better view of process economics, and scaling up.

## 2. Materials and methods

### 2.1 Synthesis and main properties of redox reactants

The priority redox system under scrutiny involves a spinel MnFe_2_O_4_ and Na_2_CO_3_ according to the reaction of Section 1.3. The preparation of the MnFe_2_O_4_/Na_2_CO_3_ mixture involved either (i) a ball-mill method involving mostly MnCO_3_ and Fe_2_O_3_, or (ii) a coprecipitation method involving MnCl_2_·4H_2_O (manganese(ii) chloride tetrahydrate) and anhydrous FeCl_3_ (iron(iii) chloride, coprecipitated with sodium hydroxide (NaOH)). Na_2_CO_3_ (>99.9% purity) was purchased from Luchi Co., Ltd. Manganese(ii) carbonate (>99.9% pure), iron(ii) oxide (>96% pure), MnCl_2_ (>99% pure) and FeCl_3_ (>98% pure), were acquired from Sigma-Aldrich Chemie GmbH.

In the ball-mill preparation,^42^ analytical grade MnCO_3_ and Fe_2_O_3_ were used without further treatment. They were mixed for 40 minutes at a molar ratio of 3 : 2 in a 1000 rpm Simoloyer CM01 mixer/mill at ambient conditions and after the addition of ethanol (96%). 5 mm diameter stainless steel balls were used at a weight ratio of 6 with respect to the reactant's mix. Ethanol was evaporated at 378 K. The dried reactant was calcined in N_2_ atmosphere at 973 K for 1 hour, and re-milled in a Retsch mill. The calcination under N_2_ is needed to activate the reactants before their first use.

For the coprecipitated MnFe_2_O_4_, an aqueous solution of 0.5 mol MnCl_2_ and 1 mol FeCl_3_ were mixed at 60 °C with continuous stirring at 250 rpm. Subsequently, a 0.64 mol NaOH solution was added. The solution was maintained at 80 °C for 1 h. The precipitates were centrifuged and wasted 5 times with distilled water at 80 °C (to remove excess NaOH and formed NaCl). After drying at 105 °C and calcination at 450 °C during 2 h, the powder was milled in a Retsch mill into fine particles (<50 μm). The chemical reaction is presented asMnCl_2_ + 2FeCl_3_ + *x*NaOH + H_2_O → MnFe_2_O_4_ + (*x* − 8)NaOH + 8NaCl + 3H_2_O

For the experiments in the vertical and solar furnaces, inert olivine was added to form a porous fixed bed, or to be able to operate the solar reactor in an isothermal fluidized mode by improving the heat transfer from the reactor wall to the fluidized bed of reactants. Malvern laser-diffraction and confirming SEM-imaging were used to determine the particle size distributions, which were mostly Gaussian with a narrow size distribution. This is illustrated in Table 2 for the feedstock particles. The near-spherical olivine particles (Mg, Fe-silicates) had a Waddell sphericity factor *ψ* of 0.8–0.9.^41^ The sphericity of all reactants was considered close to 0.84. These particles were further milled and processed into smaller particles. The pulverized mixtures had an ultrafine particle size, well below 2 μm, with smaller particles of approximately 150 nm.

**Table 2:** Average volume diameter (*d*_v_) and surface/volume diameter (*d*_sv_) of the feedstock particles

Chemicals	*d*_v_ (μm)	*d*_sv_ (μm)	*σ* (μm)
MnCO_3_	10.9	9.2	5
Na_2_CO_3_	394.5	331.3	36.5
MnCl_2_	6.4	5.9	0.5
Fe_2_O_3_	5.6	5.3	0.3
Olivine (100–150 mesh)	167.9	142.7	18.9

**Fig. 2:**
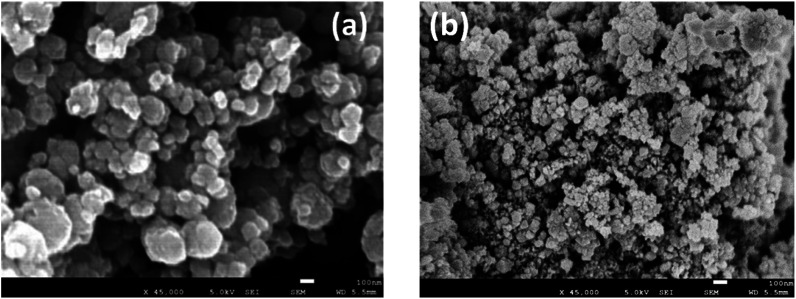
SEM images of MnFe_2_O_4_, as-synthesized at 700 °C (a) ball-milled; (b) coprecipitated.

Particle size was measured by diffractometry, and the BET surface area (m^2^ g^−1^; Brunauer–Emmett–Teller) was determined in a Micromeritics instrument by low-temperature (−196 °C) nitrogen adsorption. SEM images are shown in Fig. 2 for the prepared MnFe_2_O_4_ compound reactants (ball-milled and coprecipitated). The ball-milled reactant particles have an agglomerated particle diameter of ∼60 μm (Fig. 2a) and are composed of smaller (∼0.1 μm) grains. Higher magnifications of coprecipitated reactant (Fig. 2b) show that smaller particles of 50 to 150 nm size are obtained. The coprecipitated reactants have a higher specific surface area (132 m^2^ g^−1^) than the ball-milled reactants (4.9 m^2^ g^−1^).

### 2.2 Experimental setups and reactants

Three experimental setups were used, an electrically-heated vertical furnace for initial guidance tests, a TGA for long-duration experiments in multiple cycles, and a pilot-scale concentrated solar rig with ongoing experiments in the solar high season. Each of the experimental setups comprises the same elements.

N_2_ (carrier gas) and CO_2_ feeds are set by mass flow meters. H_2_O is added by a syringe pump. The N_2_/H_2_O (oxidation cycle) or CO_2_ (reduction cycle) flows are preheated before being added into the water splitting reactor. The reactors are either vertical furnaces or TGA cells. After the reaction, the exhaust gas is cooled and dehumidified before being sent to a GC-MS for component monitoring. CO_2_ was removed from the gas by absorption in a 1.5 g L^−1^ Ca(OH)_2_ solution. The reactors contained appropriate quantities of reactant particles. A full description of the setups and the applied experimental procedures are given in ESI-3.† Olivine was sometimes added to increase the porosity and flowability of the reactants. The ball-milled reactant was prepared by milling 7.05 g ferrite and 4.87 g Na_2_CO_3_. For coprecipitation reactant, 53.94 g of MnFe_2_O_4_ was mixed with 37.18 g of Na_2_CO_3_. The bed heights in the electrically heated furnace were 15 cm, against 25 cm in the solar reactor.

The GC-MS continuously monitored and recorded the H_2_ concentration in the oxidation step, and the O_2_ concentration in the reduction step.

## 3. Results and discussion

### 3.1 The MnFe_2_O_4_ system in the electrically heated furnace

The use of the electrically heated setup is tedious and requires frequent dismantling to obtain reactant samples. Its results however provide data for a relatively deep bed and facilitate a kinetic analysis at a fair sample scale.

**Fig. 3:**
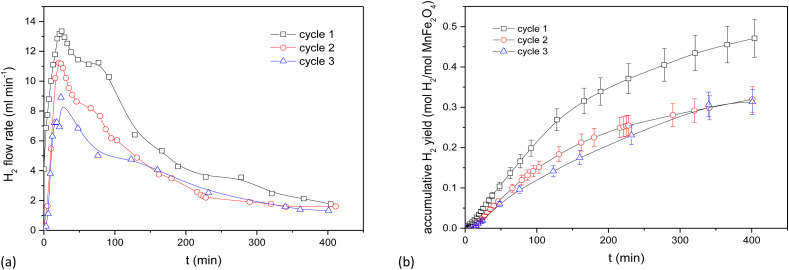
Results of the WS reaction cycle (700 °C) for co-precipitated MnFe_2_O_4_ at different oxidation time, but with a fixed reduction cycle of 3 h. (a) WS cycles of MnFe_2_O_4_ (coprecipitation). (b) Cumulative results for the WS cycles of MnFe_2_O_4_ (coprecipitation).

**Fig. 4:**
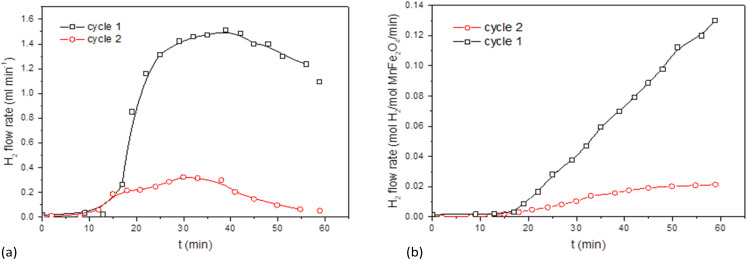
Results for ball-milled MnFe_2_O_4_ at different oxidation time, but with a fixed reduction cycle of 3 h. (a) WS cycles of MnFe_2_O_4_ (ball milling). (b) Cumulative results for the WS cycles of MnFe_2_O_4_ (ball milling).

The behaviour of the activated MnFe_2_O_4_ reactant was assessed for 3 subsequent oxidation–reduction steps each at 700 °C. The reverse step used pure CO_2_ for 3 h. Time-dependent H_2_ production values are illustrated in Fig. 3 for coprecipitated MnFe_2_O_4_, and in Fig. 4 for ball-milled MnFe_2_O_4_. The results were cumulated and expressed in mol H_2_ per mol MnFe_2_O_4_. The coprecipitated reactant significantly performs better than the reactant produced by ball milling.

Since it was seen that the ball-milled reactant had a significantly lower H_2_-yield than its coprecipitated alternative, these tests were terminated after 60 min. It was moreover evident that reduction times longer than 3 h were needed. According to Chen *et al.*,^43^ the oxygen release (step 2 of the reaction cycle) between layered Na(Mn_1/3_Fe_2/3_)O_2_ and CO_2_ is fairly slow and requires >3 h to be completed.^44,45^ This was further investigated by TGA, where it was demonstrated that the reduction step preferably requires 4.5 to 6 h. Stoichiometrically, the yield should be 0.5 mol H_2_ per mol MnFe_2_O_4_, which is only closely achieved for the coprecipitated reactant. Regrinding of the reactants between the cycles slightly increased the H_2_ yield. It was hence tentatively presumed that agglomeration or sintering of the reactant took place between cycles during different days. The effect of agglomeration can be mitigated by grinding or reactivating the reactants.

For the ball-milled reactant, the poor performance can be explained by the incomplete reaction of Fe_2_O_3_, MnCO_3_ and Na_2_CO_3_. Although some MnFe_2_O_4_ can be recovered after one cycle, some iron is segregated and forms Fe_2_O_3_ phase. The Fe_2_O_3_ phase will react with Na_2_CO_3_ and form NaFeO_2_, thus causing a decrease in H_2_ productivity between the first and second cycle due to difficulties in regeneration step. The unreacted Fe_2_O_3_ when synthesizing the ball-milled catalysts also exacerbated the decrease in H_2_ yield between cycles compared to the coprecipitated ones.

The solid–solid contact and ions transportation are equally important for the regeneration step. According to Chen *et al.*,^43^ smaller particle size with moderate crystallinity is beneficial to maintain its structure stability and can lead to a better ionic transport within the crystals than through the grain boundaries in order to finish the whole cycle. This also counts for the difference in H_2_ yield between the first two cycles. The particles smaller than 30 nm are good for the contact with Na_2_CO_3_ to release H_2_ in the first step, but hamper removing Na^+^ from the lamellar Na(Mn_1/3_Fe_2/3_)O_2_ oxide considering the low layered structure stability.

**Fig. 5:**
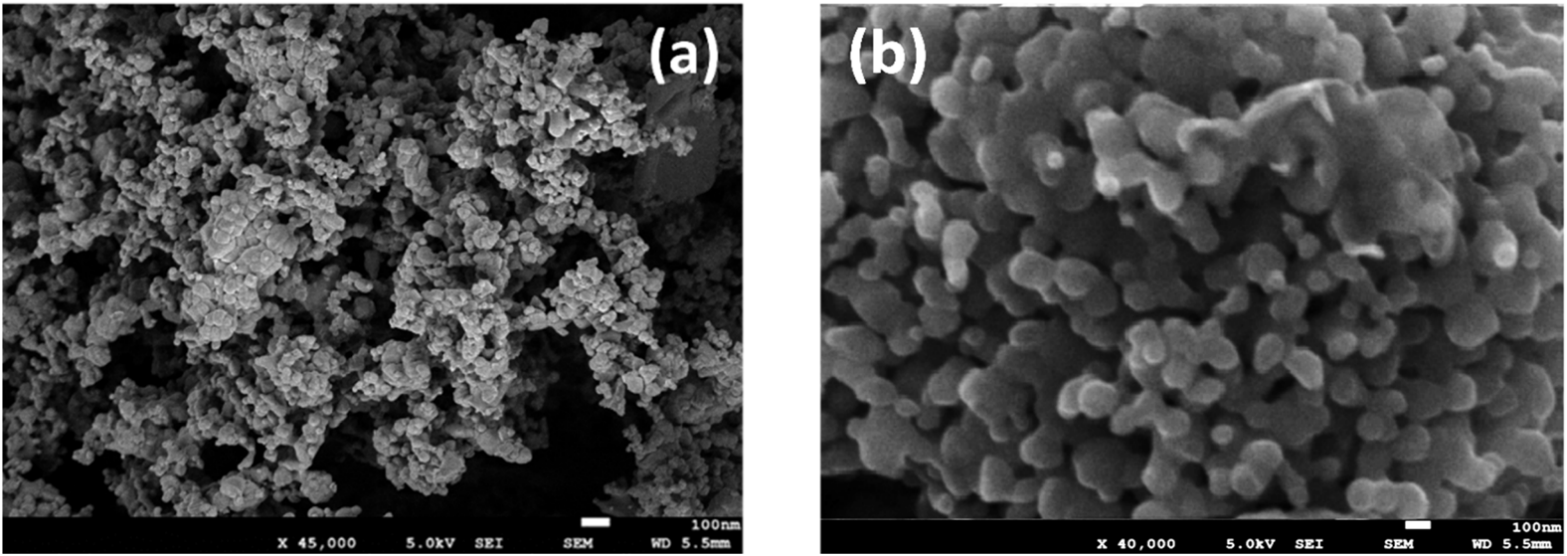
MnFe_2_O_4_ and olivine, after water splitting at 700 °C (a) ball milled; (b) coprecipitation.

The cyclic O_2_/H_2_ production was however achieved. Fig. 5 compares typical SEM images of the ball-milled and coprecipitation reactants after water splitting at 700 °C. The particles synthesized by ball-milling (Fig. 5a) formed agglomerates consisting of 0.1 μm dense grains. The co-precipitation reactant maintains a size in the order of 50–150 nm (Fig. 5b). The porous morphology of the MnFe_2_O_4_ reactants is maintained even after calcination and reaction at 700 °C.

**Fig. 6:**
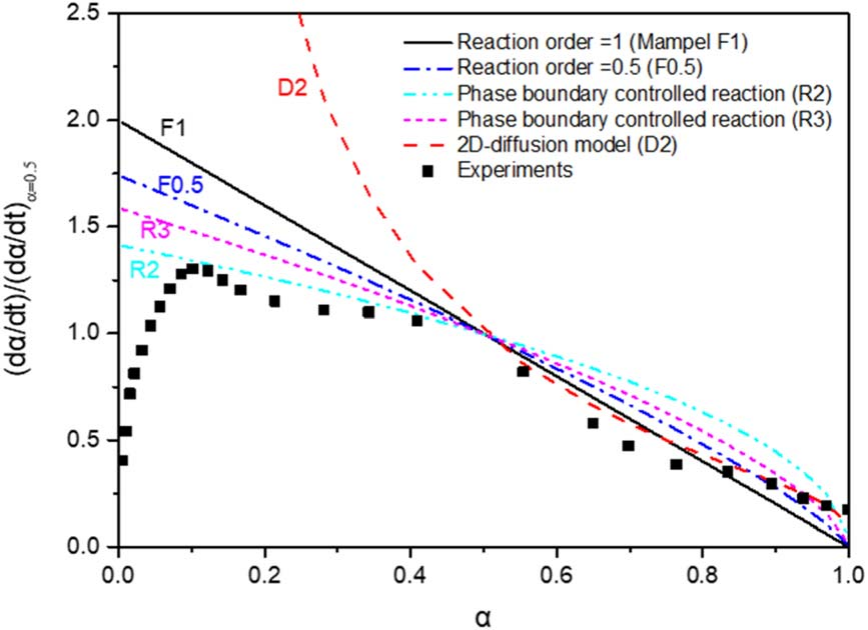
Kinetic models applied to the experimental results: *α* is the reaction fraction 
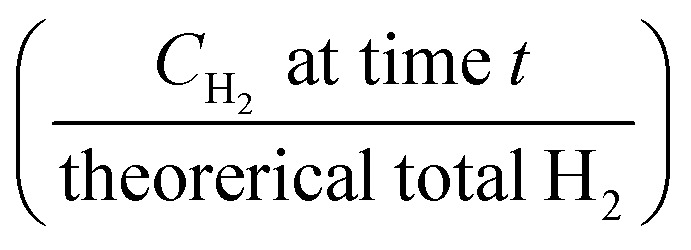
.

The time-dependent conversion allows the study of the reaction kinetics. To evaluate appropriate kinetic models,^26^ data were transformed in terms of reaction progress, *α*. These data were normalized against the conversion at *α* = 50%. All data transformations revealed similar fitting profiles, as illustrated in Fig. 6.

The MnFe_2_O_4_ water splitting is controlled by different reaction models. A 2D geometrical-contracting model (R2) seems appropriate when *α* < 0.5. For *α* > 0.5, a first-order (F1) or 2D diffusion model (D2) seems more appropriate. The morphology of Mn seems essential to the H_2_ generation reaction kinetics. This is also the case for MnO_*x*_-based water splitting reactions.^43^ An additional kinetics study was carried out based upon the TGA results, as reported below.

### 3.2 The MnFe_2_O_4_ system studied in multicycle TGA experiments

TGA experiments provide an easy follow-up of the reaction progress through the weight changes of the sample under scrutiny. With a stoichiometric weight loss during the oxidation step of 83.18% (including the added O from H_2_O), this theoretical limit forms the reference value for all the results. These results are illustrated in the following figures, obtained for a fixed oxidation duration of 3 h, but a variable reduction cycle of 3, 4.5 and 6 h.

**Fig. 7:**
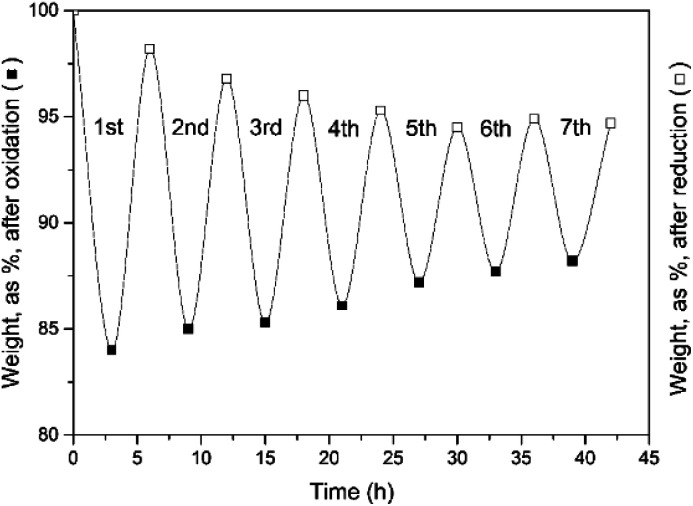
Weight evolution of the oxidation (3 h) and reduction cycles (3 h). Seven cycles were examined.

The experimental results of Fig. 7 clearly demonstrate that a 3 hour CO_2_-induced reduction cycle is insufficient to restore the initial activity of the reactant. The reaction was hence stopped after 7 cycles. This finding confirms the electrically heated furnace results.

**Fig. 8:**
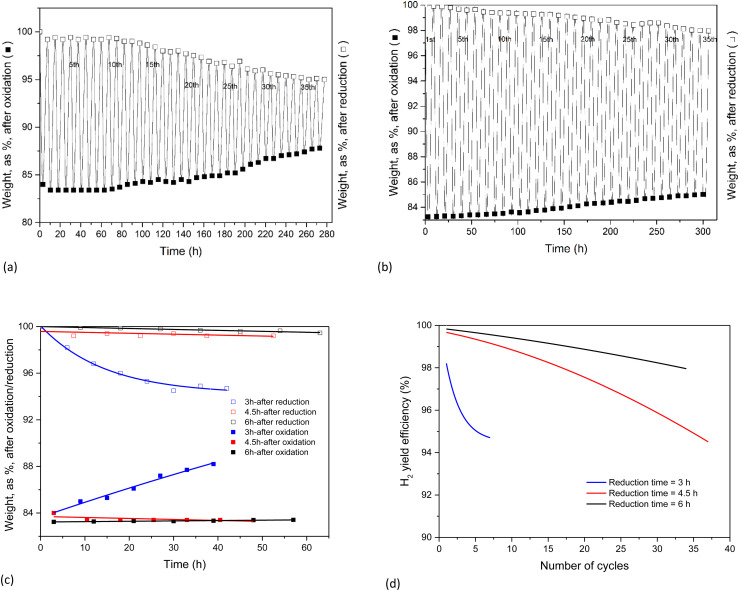
Cyclic experiments at different reduction cycle times. (a) Weight evolution of, respectively the oxidation (3 h) and reduction cycles (4.5 h). (b) Weight evolution of, respectively the oxidation (3 h) and reduction cycles (6 h). (c) The first 7 cycles at a fixed oxidation time (3 h) but variable reduction time (3, 4.5, and 6 h, respectively). (d) H_2_ yield efficiency.

Long-duration, multicycle experiments with reduction cycles of 4.5 and 6 h were performed, and the results (Fig. 8a and b) clearly demonstrated that a longer reduction cycle enhances the recyclability.

All results for the first 7 cycles (with different total cycle times) are represented in Fig. 8c.

Finally, the system H_2_ efficiency is calculated as follows, and presented in Fig. 8d:
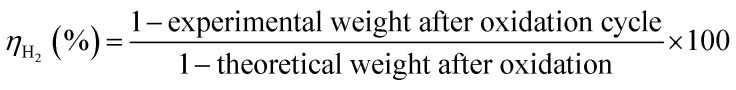


The efficiency of the combined (3 + 6) h cycles exceeded 98% after 33 cycles, again 95% only for the (3 + 4.5) h operation. It is hence recommended to combine a 3 h oxidation (H_2_ release) with 6 h of reduction (O_2_ release).

The TGA results enable the calculation of the apparent reaction rate constant (*k*). Since *α* ≫ 0.5, first-order kinetics can be applied. Table 3 summarizes the results for the given numbers of cycles and a specific reduction time (3, 4.5, and 6 h).

**Table 3:** Reaction rate constant of the oxidation step

Reaction rate constant (min^−1^)	Reduction time		
	3 h	4.5 h	6 h
Cycle 1	2.23 × 10^−2^	3.17 × 10^−2^	3.54 × 10^−2^
Cycle 6	1.68 × 10^−2^	2.72 × 10^−2^	3.07 × 10^−2^
Cycle 30	—	1.77 × 10^−2^	2.22 × 10^−2^

The reaction rate decreases with the number of cycles for a given duration of the reduction, corresponding to a progressive but limited loss of activity of the solid reactant. The rate constant however increases when the reduction time is increased. It is hence important to either extend the oxidation cycle beyond 3 h as cycling proceeds, or to re-activate the reactants more frequently by calcination and/or re-milling after a certain number of cycles.

The reaction mechanism of the water splitting has been investigated by several researchers, both in electrocatalytic and thermochemical routes. Zhou *et al.*^46,47^ studied the electrocatalytic reduction reaction (ORR), also called the hydrogen evolution reaction (HER), and oxygen evolution reaction (OER). Four elementary reaction steps are proposed, including OOH and OH species. Spin-polarized DFT simulations demonstrate that the reaction step are reported to be slow.^48^ These 4 steps were confirmed by computational screenings.^49^ The thermochemical mechanism was assessed by Chen *et al.*^43^ and Angotzi *et al.*^50^ with special emphasis on the slow oxygen release (over 3 h) of the Na(Mn_1/3_Fe_2/3_)O_2_ intermediate. In the hydrogen release step, lamellar Na(Mn_1/3_Fe_2/3_)O_2_ oxide is formed by intercalating a Na^+^ layer into two adjacent oxygen interspaces.^51^ Both intercalation of Na^+^ into adjacent oxygen interspaces and the Jahn–Teller effect lead to a more stable Na(Mn_1/3_Fe_2/3_)O_2_ oxide. The full mechanism is presented in Chen *et al.*^43^

### 3.3 Preliminary results of the MnFe_2_O_4_ system in the solar reactor

**Fig. 9:**
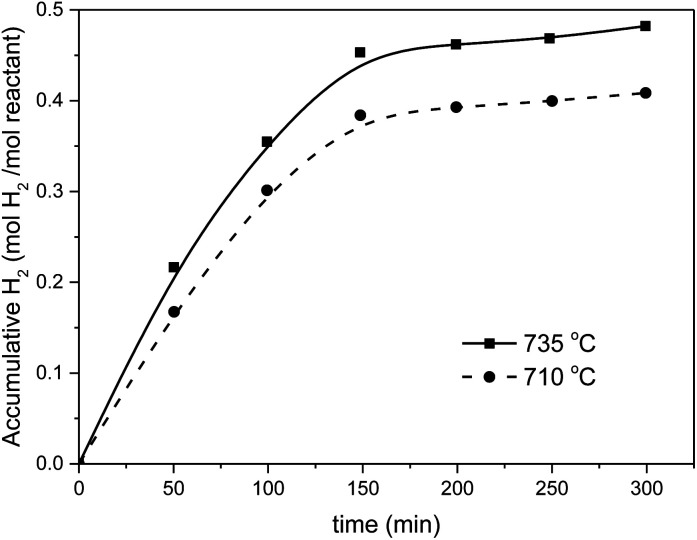
Solar H_2_ production using the MnFe_2_O_4_/Na_2_CO_3_ cycle.

Preliminary tests were performed with a solar reactor, during the first oxidation cycle. The temperature of the front reactor wall in the cavity was kept below 900 °C (thermal strength limits of the Ni/Cr construction alloy). The slow reaction of the heliostat focusing leads to temperature variations in the bed between 700 and 750 °C. Average values of 710 °C and 735 °C were maintained over a period of 5 h. The solar H_2_ production was very good and the theoretical H_2_ yield of 0.5 mol mol^−1^ was nearly achieved at 735 °C, as shown in Fig. 9.

**Fig. 10:**
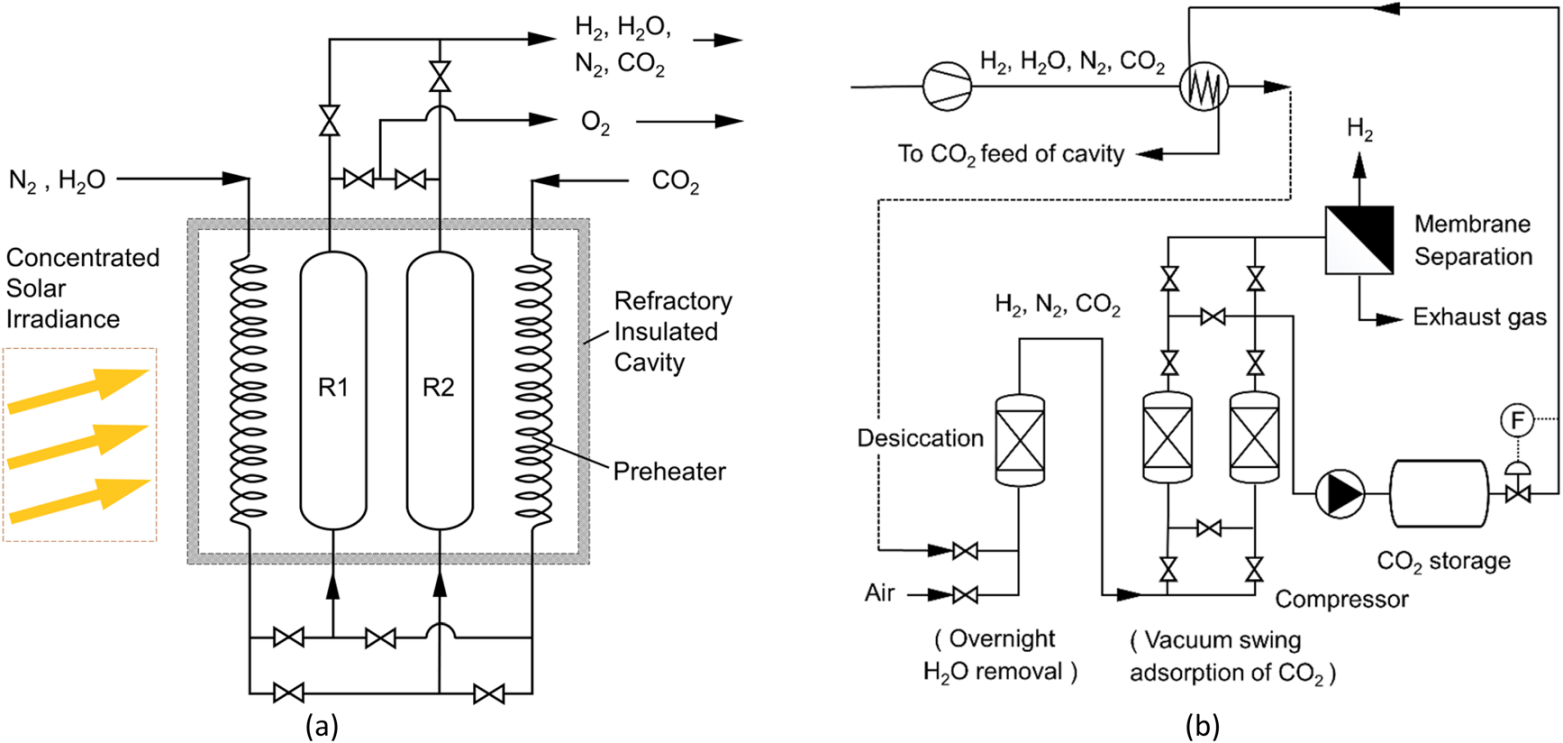
(a) Installing redox-reactors in the cavity; (b) purification cycles of H_2_. Cycle 1 oxidation (R1) progresses in parallel with the reduction cycle (R2) by appropriate value settings. The next solar day, the functions of (R1) and (R2) are reversed.

Further to the TGA observations and prior to conducting additional cycling experiments that are now taking place in the season of high direct normal irradiance (DNI), the rig was adapted to conduct oxidation and reduction steps in parallel in the single cavity, as illustrated in Fig. 10.

The H_2_ upgrading will apply a sequence of steps, involving the removal of excess H_2_O vapour; the removal of CO_2_ by vacuum swing adsorption^6^ on appropriate adsorbents, *e.g.* zeolite, activated carbon, or others;^52,53^ a membrane permeation of H_2_, *e.g.* using a Matrimid 5218 ^54^ or P84 membranes^55^ with over 95% H_2_ purity and 96% recovery. The use of sintered metal fibre filters at the reactor exhausts limits the loss of reactant.^56^

## 4. Recyclability

Results confirmed that the H_2_ production efficiency can be ≫95% provided oxidation and reduction cycles are properly performed. A conservative 95% efficiency can be accepted, as obtained both in TGA and in the solar receiver. For 0.5 mol of H_2_ per mol reactant, a maximum of ∼0.41 wt% H_2_ can be generated. Coprecipitated MnFe_2_O_4_ is purchased at approximately 500 € per ton. The ball-milled reactant can be approximately 30% cheaper.

The heating costs are not considered since the system is supposed to use excess photovoltaic or wind turbine electricity, or to operate on concentrated solar heat. Heat recovery will be maximized by good heat management. The number of required cycles (*N*_c_), to break even can be calculated for a proposed selling cost of H_2_, since:
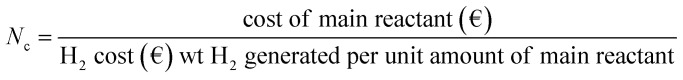


CO_2_ should be separated, stored, and used in the reverse reaction. It is also proposed to use membrane modules to produce very pure H_2_.^54,55^

The solar energy balance is currently being assessed. At 4 € per kg H_2_ and 500 € per ton of reactants, 30 cycles should be realized to break even. To reach 1 € per kg H_2_, 120 cycles should be achieved, and this is presumed possible in view of the obtained cycling results. If the cheaper ball-milled reactant could be improved, the number of required cycles to break even will be reduced.

## 5. Conclusions

This research demonstrated the high potential of water splitting by the MnFe_2_O_4_/Na_2_CO_3_ redox pair. Coprecipitated MnFe_2_O_4_ offers approximately 30% higher H_2_ productivity than its cheaper ball-milled equivalent. Experiments in an electrically-heated reactor were used to study the fundamentals, possible shortcomings, and kinetics. TGA experiments demonstrated the long-duration and multicycle (up to 37 cycles) potential. Preliminary solar reactor experiments confirm the >95% H_2_ efficiency: these solar experiments are ongoing in a joint oxidation/reduction parallel reactor, installed in the solar cavity. The CO_2_ reaction time should be approximately 6 h to achieve a good reduction. Tentative cost calculations showed a break-even operation for 30 consecutive cycles at H_2_ prices of 4 € per kg H_2_. At least 120 cycles before reactant regeneration will reduce the H_2_ production cost to ∼1 € per kg H_2_. This implies the use of a cheap energy supply, and the complete reuse of CO_2_ in the reduction reaction. The results certainly foster a further improvement of the system.

## Author contributions

R. Dewil and J. Baeyens supervised the research. Y. Deng, S. Li, and M. Yang designed and performed the experiments. L. Appels and H. Zhang participated in the data analyses and discussions. Y. Deng, R. Dewil, and J. Baeyens wrote the paper. All authors discussed the results and commented on the manuscript.

## Conflicts of interest

There are no conflicts to declare.

## Supplementary Material
